# Metabolic consequences of inflammatory disruption of the blood-brain barrier in an organ-on-chip model of the human neurovascular unit

**DOI:** 10.1186/s12974-016-0760-y

**Published:** 2016-12-12

**Authors:** Jacquelyn A. Brown, Simona G. Codreanu, Mingjian Shi, Stacy D. Sherrod, Dmitry A. Markov, M. Diana Neely, Clayton M. Britt, Orlando S. Hoilett, Ronald S. Reiserer, Philip C. Samson, Lisa J. McCawley, Donna J. Webb, Aaron B. Bowman, John A. McLean, John P. Wikswo

**Affiliations:** 1 Department of Physics and Astronomy, Vanderbilt University, Nashville, TN 37235 USA; 2 Vanderbilt Institute for Integrative Biosystems Research and Education, Vanderbilt University, 6301 Stevenson Center, Nashville, TN 37235 USA; 3 Department of Chemistry, Vanderbilt University, Nashville, TN 37235 USA; 4 Center for Innovative Technology, Vanderbilt University, Nashville, TN 37235 USA; 5 Department of Biological Sciences, Vanderbilt University, Nashville, TN 37235 USA; 6 Vanderbilt Institute of Chemical Biology, Vanderbilt University, Nashville, TN 37232 USA; 7 Department of Biomedical Engineering, Vanderbilt University, Nashville, TN 37235 USA; 8 Department of Pediatrics, Vanderbilt University Medical Center, Nashville, TN 37232 USA; 9 Vanderbilt Kennedy Center, Vanderbilt University Medical Center, Nashville, TN 37203 USA; 10 Vanderbilt Brain Institute, Vanderbilt University, Nashville, TN 37232 USA; 11 Department of Cancer Biology, Vanderbilt University, Nashville, TN 37232 USA; 12 Department of Neurology, Vanderbilt University Medical Center, Nashville, TN 37232 USA; 13 Department of Biochemistry, Vanderbilt University, Nashville, TN 37232 USA; 14 Department of Molecular Physiology and Biophysics, Vanderbilt University, Nashville, TN 37232 USA

**Keywords:** Lipopolysaccharide, Cytokine, Tight junctions, IL-1β, TNF-α, MCP1,2, Brain-on-chip, Micro-organ, Mass spectrometry, Metabolomics

## Abstract

**Background:**

Understanding blood-brain barrier responses to inflammatory stimulation (such as lipopolysaccharide mimicking a systemic infection or a cytokine cocktail that could be the result of local or systemic inflammation) is essential to understanding the effect of inflammatory stimulation on the brain. It is through the filter of the blood-brain barrier that the brain responds to outside influences, and the blood-brain barrier is a critical point of failure in neuroinflammation. It is important to note that this interaction is not a static response, but one that evolves over time. While current models have provided invaluable information regarding the interaction between cytokine stimulation, the blood-brain barrier, and the brain, these approaches—whether in vivo or in vitro—have often been only snapshots of this complex web of interactions.

**Methods:**

We utilize new advances in microfluidics, organs-on-chips, and metabolomics to examine the complex relationship of inflammation and its effects on blood-brain barrier function ex vivo and the metabolic consequences of these responses and repair mechanisms. In this study, we pair a novel dual-chamber, organ-on-chip microfluidic device, the NeuroVascular Unit, with small-volume cytokine detection and mass spectrometry analysis to investigate how the blood-brain barrier responds to two different but overlapping drivers of neuroinflammation, lipopolysaccharide and a cytokine cocktail of IL-1β, TNF-α, and MCP1,2.

**Results:**

In this study, we show that (1) during initial exposure to lipopolysaccharide, the blood-brain barrier is compromised as expected, with increased diffusion and reduced presence of tight junctions, but that over time, the barrier is capable of at least partial recovery; (2) a cytokine cocktail also contributes to a loss of barrier function; (3) from this time-dependent cytokine activation, metabolic signature profiles can be obtained for both the brain and vascular sides of the blood-brain barrier model; and (4) collectively, we can use metabolite analysis to identify critical pathways in inflammatory response.

**Conclusions:**

Taken together, these findings present new data that allow us to study the initial effects of inflammatory stimulation on blood-brain barrier disruption, cytokine activation, and metabolic pathway changes that drive the response and recovery of the barrier during continued inflammatory exposure.

**Electronic supplementary material:**

The online version of this article (doi:10.1186/s12974-016-0760-y) contains supplementary material, which is available to authorized users.

## Background

Recent research has shown that systemic infection and inflammation not only affect multiple organs in the body but also the central nervous system (CNS). An excellent example of this is maternal immune activation, which increases the risk of neurological disorders in the gestating fetus [1–7]. Previous studies have determined that traumatic brain injuries and cancer can activate the immune system and affect the CNS [8, 9]. These reports support the idea that substances created by peripheral immune responses are crossing the blood-brain barrier (BBB) and affecting CNS function. Furthermore, immune activators such as lipopolysaccharide (LPS) have been shown to impair intestinal barrier function, and in neural tissue culture, LPS has been reported to induce cytokine activation and cell damage [10–12]. Recent animal studies have illustrated that LPS exposure in pregnant mice elevates fetal IL-6 and perturbs fetal brain development [7, 13]. Other data collected in rodents suggest that the BBB is relatively impermeable to LPS [14]. While the rodent work is compelling, it can be difficult to reconcile with human postmortem studies [15, 16] and observations made in cell culture, which seem to indicate a much more pronounced effect of LPS on the BBB [17]. One limitation of these studies is that due to the high cost of sample collection from both animals and human patients, often only a single time point after a single, short-duration exposure is analyzed. Systemic immune activation is a process that develops over time [18, 19], and thus, BBB function should be assessed in a dose- and time-dependent manner. In addition to LPS directly crossing the human BBB, the cytokines it induces in the vasculature that comprise the BBB certainly can cross the barrier and affect its function [20–22].

To aid our further understanding of how the BBB responds to immune activation, we have developed a novel dual-chamber microfluidic device that models BBB function. It utilizes human primary cells and enables flow and sample collection from both compartments (brain and vascular side) separated by a barrier [23]. By using this novel technology, we are able to continually perfuse the vascular side of the BBB model with LPS or a cytokine cocktail and to collect effluent samples from before and during the course of exposure. In these studies, our data suggest that LPS has a time-dependent effect on BBB permeability, cytokine activation, and metabolic activity, and we observe a robust metabolic pathway activation response using a mixture of TNF-α, IL-1β, and MCP1,2.

## Methods

### NeuroVascular Unit microfluidic device

The NeuroVascular Unit (NVU) was fabricated by the Vanderbilt Institute for Integrative Biosystems Research and Education (VIIBRE) Microfabrication Core. The basic design is a two-chamber system wherein the chambers are constructed of polydimethylsiloxane (PDMS) and divided by a porous 0.2-μm polycarbonate membrane (Sigma-Aldrich, St. Louis, MO, USA). Each chamber has its own inlet and outlet for perfusion. The device can be perfused in any orientation, so that cells seeded into a chamber can be induced by gravity to adhere to one side of the porous membrane and by inverting the device and seeding the opposite side with a different cell type, they can be grown in opposition to one another to form the BBB [23]. As previously described [23], the bottom/vascular chamber is 2.9 μL in volume, with uniform shear forces across the chamber, while the top/brain chamber is 18 μL in volume. The top perfusion layer is attached to the brain chamber and allows a mild media exchange without the introduction of shear stress onto the neurons and co-differentiating astrocytes (cultured within the collagen) and the astrocytes and pericytes that are adhered to the barrier.

### Cell culture

Cell culture was carried out as previously described [23]. In brief, cells used to establish our NVU BBB model include primary human brain-derived microvascular endothelial cells (hBMVEC) from Applied Cell Biology (Kirkland, WA, USA) and pericytes and astrocytes from ScienCell (Carlsbad, CA, USA) and ATCC (Manassas, VA, USA), respectively. Before endothelial cells were introduced into the lower vascular chamber of the NVU, the device was coated with laminin at 9.6 μg/mL for 24 h at 37°C. On day 0, hBMVECs were loaded into the vascular chamber (1 × 10^6^ cells/mL), followed by device inversion to allow cell attachment to the membrane. At day 1, media was perfused at 2 μl/min, and the hBMVECs were allowed to grow for 12 days to reach confluence and establish tight junctions. On day 12, the device was returned to its original orientation and a 2:1 mix of primary human astrocytes and pericytes was loaded into the upper brain chamber and allowed 1–2 days to reach confluence. On day 14, collagen gel containing 4 million human induced pluripotent stem cell (hiPSC)-derived human cortical neurons and co-differentiating astrocytes per milliliter was loaded on top of the astrocytes and pericytes. The hiPSC-derived neurons and co-differentiating astrocytes were differentiated to days 95–200 via dual-SMAD inhibition followed by terminal differentiation by adapting methods described elsewhere, except that LDN was used at 4 μM [24–27]. The collagen gel was given 2 h to solidify before restarting perfusion of the brain chamber. For the first 3 days after the neurons were added, the brain chamber was perfused with media containing Rho-associated coiled-coil kinase (ROCK) inhibitor (10 μM, Tocris) Y-27632 (Sigma-Aldrich, St. Louis, MO, USA) to help the neurons survive the stress of replating [28–31]. Once the ROCK inhibitor was no longer needed, we used media without it, and the NVU was ready for testing.

### LPS and cytokine cocktail exposure and sample collection

LPS was purchased from Sigma-Aldrich (St. Louis, MO, USA) and applied to the vascular chamber at 100 μg/mL, which is a concentration that has previously been shown to disrupt tight junctions and increase intestinal permeability [32]. Effluent samples were collected from both the vascular and brain chambers of the NVU at three time points: before exposure (denoted as 0), 6 h after exposure, and 24 h after exposure. For the cytokine cocktail, 100 ng/ml of TNF-α, IL-1β, and MCP1,2 (Sigma-Aldrich, St. Louis, MO, USA) were diluted in vascular media [23] and applied to the vascular chamber only for 24 h.

### Live/dead evaluation

To evaluate the DNA integrity of the cells, a live/dead assay kit (Cat. No. L3224, Fisher, Waltham, MA, USA) was used according to the manufacturer’s instructions using 1 μM of calcein AM (i.e., live stain indicator) and 2 μM of ethidium homodimer-1 (i.e., dead stain indicator), as previously described [33]. Cells that are stained with red are considered unhealthy. All live/dead images were taken from the same frame with different excitation wavelengths.

### FITC-dextran diffusion and transendothelial electrical resistance

Fluorescein isothiocyanate-dextran (FITC-dextran) of 10 kDa (Sigma-Aldrich, St. Louis, MO, USA) was prepared at 1 μg/mL (100 nM) in cell culture media for diffusion testing. As previously described [23], the vascular compartment of the NVU was perfused with 10 kDa solution for 23 h. In our original protocol [23] at 23 h, the flow on both sides (vascular and brain chambers) was stopped for 1 h, allowing the dextran to diffuse across the BBB and accumulate in the brain compartment. After 1 h, perfusion of both chambers was restarted and individual effluents were collected for fluorescent intensity analysis using a plate reader (TECAN M1000). We have shown that the stop-flow was not necessary and now collect fluid for 1 h of continuous perfusion with FITC-dextran. By measuring FITC-dextran diffusion across the membrane, we were able to evaluate the effectiveness of the BBB [34].

Transendothelial electrical resistance (TEER) measurements were performed using our custom-built multi-frequency impedance analyzer based on an AD5933 chip (Analog Devices, Nashua, NH, USA) utilizing a four-probe approach [35]. The largest changes in impedance as a function of BBB maturation were observed at 15 kHz, and all TEER measurements used in this study to evaluate the effect of LPS on BBB function are reported at that frequency.

### Tight junction staining

Fluorescent labeling of the tight junctions was evaluated using a Zeiss Axiovert 200 automated microscope equipped with a CoolSnap CCD camera. Collected images were analyzed with ImageJ. Tight junction staining was conducted as detailed in [36], using ZO-1 and claudin-5 (Invitrogen, Grand Island, NY, USA) directly conjugated to Alexa 488 [37]. Briefly, greyscale measurements of the border between cell plasma membranes were taken for ten different cell-to-cell junctions in four different plates, and an average intensity was derived for each culture condition [23].

### Cytokine ELISA

Effluent samples taken before 6 h and after 24 h of LPS exposure were collected and diluted 1:3 for cytokine analysis using either the V-Plex Human Cytokine Kit (Meso Scale Discovery, Rockville, MD, USA) or Quantikine enzyme-linked immunosorbent assay (ELISA) for TNF-α and IL-1β (R&D Systems, Inc., Minneapolis, MN, USA). Sample preparation was carried out as described previously [38].

### Data analysis

The statistical analysis of the mass spectrometry data is described within the section on metabolite data processing and analysis. All other data were analyzed in a blinded fashion using GraphPad software. Analysis of variance (ANOVA) models were used to analyze the data and contained one between-subjects variable, such as “treatment” and “trial number” (e.g., before and after drug treatment). The appropriateness of ANOVA models was evaluated by considering the distributional properties of the variables studied and by the adequacy of the homogeneity of variance assumption. The Greenhouse-Geisser (or Huynh-Feldt) adjustment was used for all within-subjects effects containing more than two levels in order to protect against violations of the sphericity/compound symmetry assumptions when a repeated measure ANOVA was used.

### Metabolite extraction

All solvents used for metabolite extraction (methanol, water, acetonitrile, and formic acid) were liquid chromatography (LC)-mass spectrometry (MS) grade (Fisher Scientific, Fair Lawn, NJ, USA). Metabolites were extracted from NVU media using a MeOH:H_2_O (80:20, *v*:*v*) solvent extraction mixture. A volume of 500 μL of cold (−20°C) extraction mixture was added to each 60 μL aliquot of media, vortexed for 30 s, and incubated at −80°C overnight to precipitate proteins. After incubation, samples were cleared by centrifugation at 15,000 rpm for 15 min, and the resulting supernatant was removed and evaporated to dryness in a vacuum concentrator. Dried extracts were reconstituted in 60 μL of C_18_ reconstitution solvent mixture containing 98:2 (*v*:*v*) H_2_O:ACN with 0.1% formic acid for reverse phase analysis, followed by centrifugation for 5 min at 15,000 rpm to remove insoluble debris. Quality control samples were prepared by combining equal volumes (10 μL) of each sample type (experimental design and sample workflow are shown in Additional file 1).

### Mass spectrometry

Ultraperformance liquid chromatography-ion mobility-mass spectrometry (UPLC-IM-MS) and data-independent MS acquisition with simultaneous analysis of molecular fragmentation (MS^E^) were performed on a Waters Synapt G2 HDMS (Milford, MA, USA) mass spectrometer equipped with a Waters nanoAcquity UPLC system and autosampler (Milford, MA, USA), as previously described [39]. Metabolites were separated on a reverse phase 1 mm × 100 mm HSS T3 C_18_ column packed with 1.8-μm particles (Waters, Milford, MA, USA) held at 45°C. Liquid chromatography was performed using a 30-min gradient at a flow rate of 75 μL min^−1^ using mobile phase A (0.1% formic acid in H_2_O) and mobile phase B (0.1% formic acid in ACN). The following elution gradient was used for analysis: 0 min, 99% A; 1 min, 99% A; 10 min, 40% A; 20 min, 1% A; 22 min, 1% A; 25 min, 99% A.

High-definition MS^E^ (HDMS^E^) analyses were run using resolution mode, with a capillary voltage of 2.75 kV, source temperature at 100°C, sample cone voltage at 30 V, extraction cone voltage at 5 V, source gas flow of 400 mL min^−1^, desolvation gas temperature of 325°C, He cell flow of 180 mL min^−1^, and an ion mobility (IM) gas flow of 90 mL min^−1^. The data were acquired in positive ion mode from 50 to 2000 Da with a 1-s scan time; leucine enkephalin was used as the lock mass (*m/z* 556.2771). All analytes were analyzed using MS^E^ with an energy ramp from 10 to 40 eV and an injection volume of 5 μL [40]. (For the work flow, see Additional file 1.)

### Metabolite data processing and analysis

The acquired UPLC-IM-MS^E^ data were imported, processed, normalized, and interpreted in Progenesis QI v.2.1 (Nonlinear Dynamics, Newcastle, UK). Each UPLC-IM-MS^E^ data file was imported as an ion intensity map (used for visualization in both m/z and retention time dimensions), followed by retention time alignment and peak picking. Peak picking was performed on individual aligned runs by matching peaks in an aggregate data set that was created from all aligned runs. Following peak picking, the features (retention time and m/z pairs) were reduced using both adduct ([M + H]^+^, [M + Na]^+^, [M + K]^+^, etc.) and isotope deconvolution. Data were normalized to all compounds as an abundance ratio between the run being normalized and a reference run. Statistically significant changes were identified using multivariate statistical analysis, including principal component analysis (PCA), and *p* values were generated using ANOVA or pairwise comparison. Volcano plots (log_2_ fold change vs. −log_10_ *p* value) were generated for basal conditions (no LPS treatment) vs. 100 μg/mL LPS stimulation after either 6 or 24 h. Four biological replicates (NVU) and two technical replicates from each sample type were used to calculate the fold change and *p* value, and features were considered differentially expressed only if they met both criteria of fold change ≥|2| and significance (*p* ≤ 0.05); we have termed this list “prioritized metabolites”. Feature lists generated from different individual comparisons were visually compared using Venn diagrams generated by the Venny software package [41]. Statistically significant metabolites or compounds were assigned tentative structural identifications using accurate mass measurements (<10 ppm error) and isotope distribution by searching the Human Metabolome Database (HMDB) [40], METLIN [42], MassBank [43], and the NIST 14 Tandem Database and Search Program of the National Institute of Standards and Technology [44]. Following tentative structural identifications, further data processing was performed by removing metabolites associated with drugs, plants, food, and microbial origin. Metabolite peak identifications were putatively assigned using product ions observed in the fragment ion spectra analyzed in HDMS^E^ mode by searching the aforementioned databases. Ion mobility separations were used to isolate precursor ions and correlate product ions [45].

### Metabolic activity network mummichog analysis

Metabolomics pathway analysis was performed by *mummichog* software 1.0.5 using default parameters. Compound ions measurement files exported from Progenesis QI analysis software were used to generate the *mummichog* input files*. mummichog* tested the enrichment of input metabolites against random data resampled from the list of compounds by permutations and produced an empirical *p* value for known biological pathways. Input metabolites in the significant pathways (*p* value ≤0.05) were linked in a network figure by known metabolic pathways [46].

## Results

### Inflammatory signals and cell viability

**Fig. 1:**
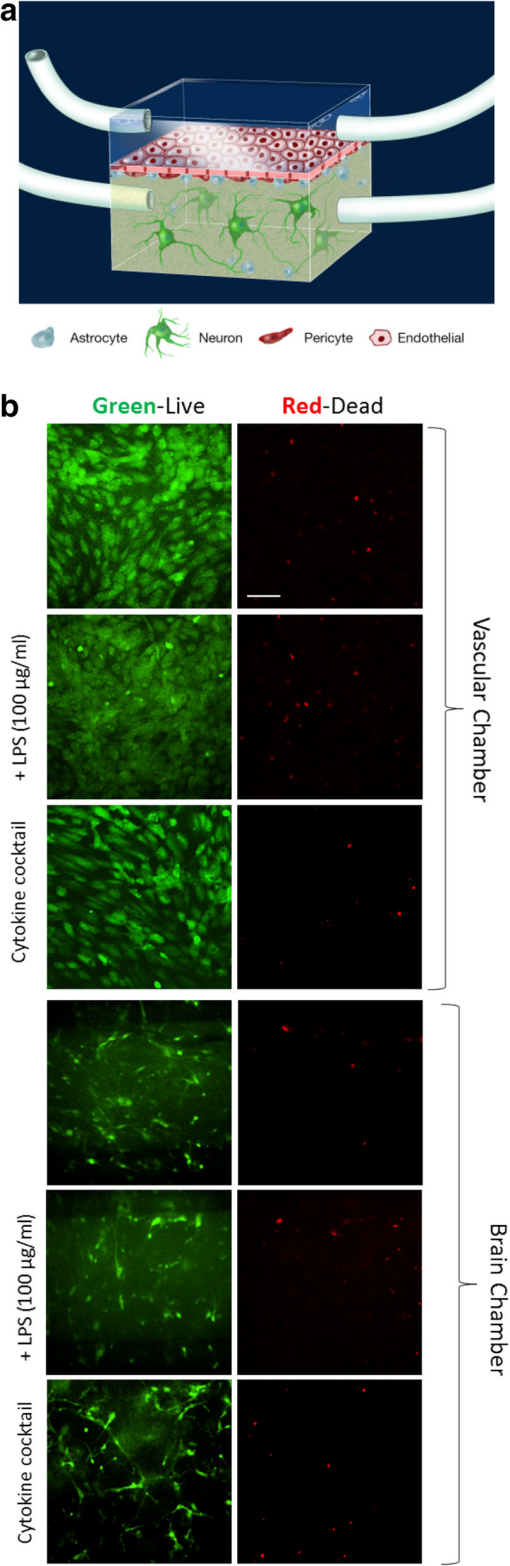
NeuroVascular Unit (NVU) layout and live/dead staining before and after inflammatory stimulation. **a** Artistic rendering of the NVU and the cells contained within. **b** Vascular chamber live/dead and **c** brain chamber with and without 24-h exposure to 100 μg/mL LPS or cytokine cocktail of 100 ng/ml TNF-α, IL-1β, and MCP1,2. *Green* and *red channels* are taken from the same frame. *Scale bar* is 200 μm

Although it has been well established that exposure to LPS induces cytokine responses [16, 32], and in the case of other organ systems that LPS exposure has been linked to reduced tight junction protein expression [17], relatively little is known about its effects on the BBB and how this compares to the direct cytokine exposure that LPS is supposed to induce. To study the effects of inflammatory signals on BBB function, we leveraged novel microfluidic technology in a dual-chambered device, creating a system that contains the relevant cell types for BBB formation and enables these cells to form the barrier in the presence of flow and a differential serum concentration from the vascular to the brain side of the device [23]. Once the brain-derived endothelial cells, astrocytes, and pericytes have had a chance to form the basis for the BBB, hiPSC-derived cortical neurons and co-differentiating astrocytes are suspended in a collagen gel and loaded on top of the astrocytes and pericytes. This entire component is what we call the NeuroVascular Unit (NVU), which previously has been shown to restrict diffusion of small molecules and facilitate active transport [23]. Having established our cellular model of the BBB (Fig. 1a), we then sought a concentration of LPS and cytokine cocktail that, while activating the system, would not cause cell death. We wanted the highest concentration that did not increase cell death from control. Over the dose ranges tested (25 to 100 μg/ml), we found that 100 μg/mL showed no cell death in either the vascular chamber containing endothelial cells (Fig. 1b) or in the brain chamber containing pericytes, astrocytes, and neurons (Fig. 1c), as assessed by live/dead staining after 24 h of LPS or cocktail exposure.

### Blood-brain barrier transport of inflammatory signals

**Fig. 2:**
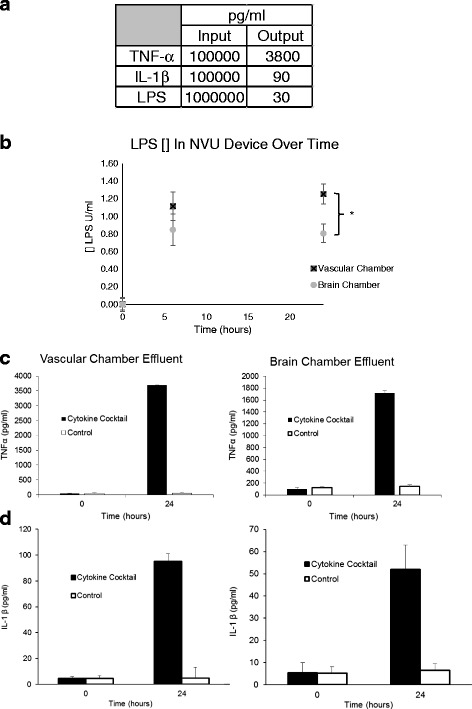
Blood-brain barrier transport of inflammatory signals. **a** Concentration in pg/ml in effluent going into an empty bioreactor and effluent coming out after 24 h of continuous perfusion at 2 μl/min. **b** Concentration of LPS in vascular and brain chambers over time. At 24 h, the brain is significantly lower than vascular (*p* = .0035, *N* = 8). **c** ELISA of TNF-α in NVU before and after treatment of the vascular side only with cytokine cocktail containing 100 ng/ml TNF-α, IL-1β, and MCP1,2 shows 31% transport of TNF-α to neuronal/brain chamber. **d** ELISA of IL-1β in NVU before and after treatment of the vascular side only with cytokine cocktail containing 100 ng/ml TNF-α, IL-1β, and MCP1,2 shows 36% transport of IL-1β to neuronal/brain chamber (*N* = 8)

The custom microfluidic device used to generate the organ-on-chip model of the BBB and the NVU consists primarily of PDMS, a polymer with many desirable features for cell culture, being easily molded, cell-compatible, and gas-permeable, to name a few. We note, however, that it also has been shown in tests to absorb small hydrophobic molecules [47–49]. To know the actual dose of inflammatory signals reaching our NVU BBB, we first looked at absorption in an empty device (Fig. 2a), and from these findings, we observed that while we started with relatively high concentrations, final exposure was much closer to physiologic ranges seen in patients at the onset of severe sepsis (~300 pg/ml) [50], with the greatest loss being LPS itself. When we next examined the transport of LPS vs. cocktail across the BBB in the NVU, we saw that the percentage of LPS transport was 46% (Fig. 2b) and that of TNF-α and IL-1β was 31 and 35%, respectively (Fig. 2c,d). These data indicate that both LPS and cytokines it often stimulates cross the barrier with relatively high efficacy.

### Inflammatory stimulation effects on blood-brain barrier integrity

**Fig. 3:**
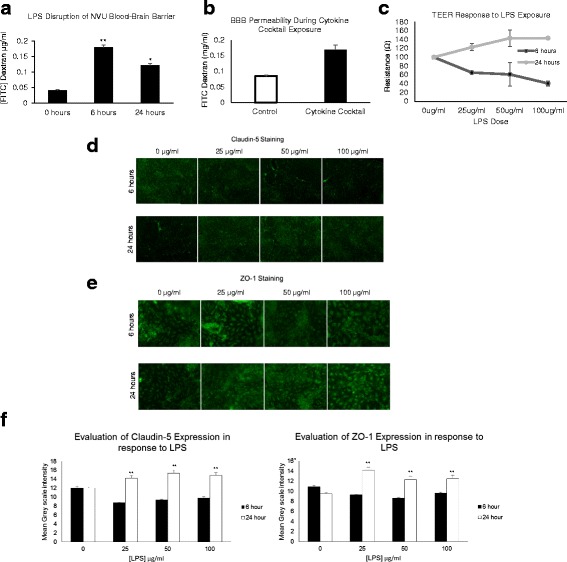
FITC-dextran diffusion across the BBB and tight junction staining in response to inflammatory stimulation over time. **a** FITC-dextran diffusion across the BBB is significantly increased at 6 h (*p* = .05, *N* = 7) and reduced from the 6-h point at 24 h (*p* = .05, *N* = 7). **b** FITC-dextran diffusion across the BBB is significantly increased 24 h after start of cytokine cocktail exposure (*p* = .01, *N* = 4). **c** TEER of dose- and time-dependent effect with LPS response. **d** Claudin-5 staining of tight junctions shows dose- and time-dependent response, with staining decreasing at early time points with dose and increasing at later time points with dose. **e** ZO-1 staining of tight junctions shows dose- and time-dependent response, with staining decreasing at early time points with dose and increasing at later time points with dose. **f** Mean grayscale intensity of the images such as those in **b** and **c** for Claudin-5 and ZO-1 release in response to LPS concentration over time

To measure directly how BBB permeability was changing over time in response to LPS, we determined the diffusion of 10-kDa FITC-dextran across the NVU BBB. As was expected, at time 0, before LPS exposure, diffusion was extremely low; however, after 6 h of exposure to LPS introduced on the vascular side, diffusion was significantly increased four times over the control at time 0 (*p* = .0001, *N* = 7). Interestingly, at 24 h, diffusion was reduced from its level at 6 h but had not returned to pre-exposure levels (Fig. 3a). Having thus established a time-course effect of LPS on membrane resistance and barrier diffusion, we then assessed how this compared to direct cytokine stimulation and found the effect on BBB permeability to be similar (Fig. 3b). We also considered how transendothelial resistance changed as a function of LPS and time. During early exposures to LPS for six continuous hours, TEER measurements suggest that the permeability of the NVU BBB does increase as a function of time and dose, as observed by a reduction in resistance. An inverse response is observed after 24 h of exposure to LPS, however; i.e., TEER measurements show an increase in membrane resistance as a function of time and dose (Fig. 3c).

Additional experiments showed the expression and localization of tight junction proteins as a response to dose and time of LPS exposure. For both claudin-5 and ZO-1, we saw a dose-dependent reduction in levels of expression at 6 h of LPS exposure and an increase after 24 h (Fig. 3d, e). When the staining intensity was quantified over multiple samples, we observed a significant decrease in both tight junction proteins at 6 h (−12% ZO-1, −18% Clad-5 (*p* ≤ .03, *N* = 10)) and a significant increase at 24 h (31% ZO-1, Clad-5 22% (*p* ≤ .03, *N* = 10)) for all concentrations tested (Fig. 3f shows the quantitation of the staining). These results indicate that the changes in the NVU BBB as a result of LPS exposure directly correlate with tight junction protein expression. Taken together, these findings argue that although the BBB may initially become more permeable when exposed to this foreign immunogen, over time it begins to block LPS passage—this is related to tight junction protein expression. These results also showed similar disruption of the BBB by cytokines, as was seen with LPS, suggesting overlapping mechanisms.

### Cytokine activation in BBB model as a result of LPS stimulation

**Fig. 4:**
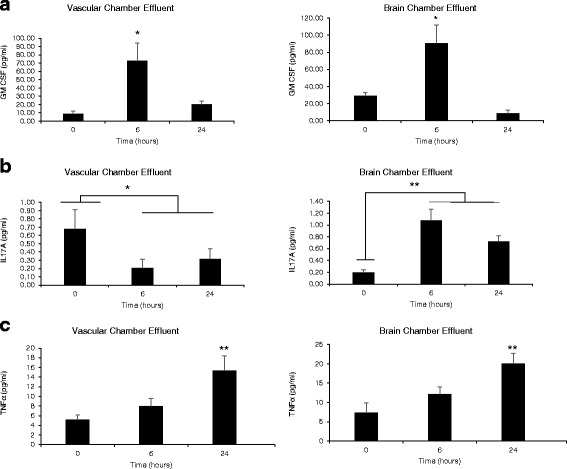
ELISA quantification of cytokine response to LPS stimulation over time. **a** Vascular chamber and brain chamber both elevate GM-CSF at 6 h but not 24 h (*p* = .02, *p* = .016, *N* = 7). **b** Vascular chamber decreases IL-17A at 6 and 24 h (*p* = .05, *p* = .05, *N* = 7), whereas brain chamber increases IL-17A at 6 and 24 h (*p* = .001, *p* = .002, *N* = 7). **c** Vascular chamber and brain chamber show similar time-dependent elevations in TNF-α (*p* = .01, *p* = .01, *N* = 7)

The link between LPS exposure and cytokine activation has been well established in both cell culture and animal models. Despite this wealth of data, few studies have investigated cytokine response as a function of time or changes in barrier permeability. Using 50 μL of effluent collected from the vascular and brain side of our NVU devices at 0, 6, and 24 h of LPS exposure, we ran ELISA detection for a battery of cytokines (GM-CSF, IL12-p40, IL-15, IL-16, IL-1a, IL-5, IL-17A, TNF-b, VEGF, TNF-α, IL-1β ). Interestingly, of the 11 cytokines investigated, over half exhibited significant changes in one or more of the chambers and time points under investigation; however, different patterns of cytokine release and membrane permeability were observed when individual cytokines were studied alone. For example, the cytokine granulocyte-macrophage colony-stimulating factor (GM-CSF) known to be active in BBB disruption [51] showed a pattern of release in both vascular and brain chambers very similar to that seen in FITC-dextran diffusion, wherein release was highest at 6 h and markedly reduced at 24 h (Fig. 4a). However, if we look at a cytokine such as IL-17A, which is known to stimulate neurite outgrowth [52], its release levels in the vascular chamber were significantly reduced at 6 and 24 h, but increased at these time points in the brain chamber (Fig. 4b). Finally, the canonical cytokine TNF-α, which is known to be part of the inflammatory response to BBB disruption [53, 54], showed an increase in release over time in both the vascular and brain chambers (Fig. 4c). (For a complete list of all the cytokines and their fold changes, see Additional file 2.) From these findings, we perceive a complex array of cytokine changes that occur over time in the BBB, and these changes may result in the observed alterations of barrier permeability.

### Metabolic response of BBB model to LPS and cytokine cocktails

**Fig. 5:**
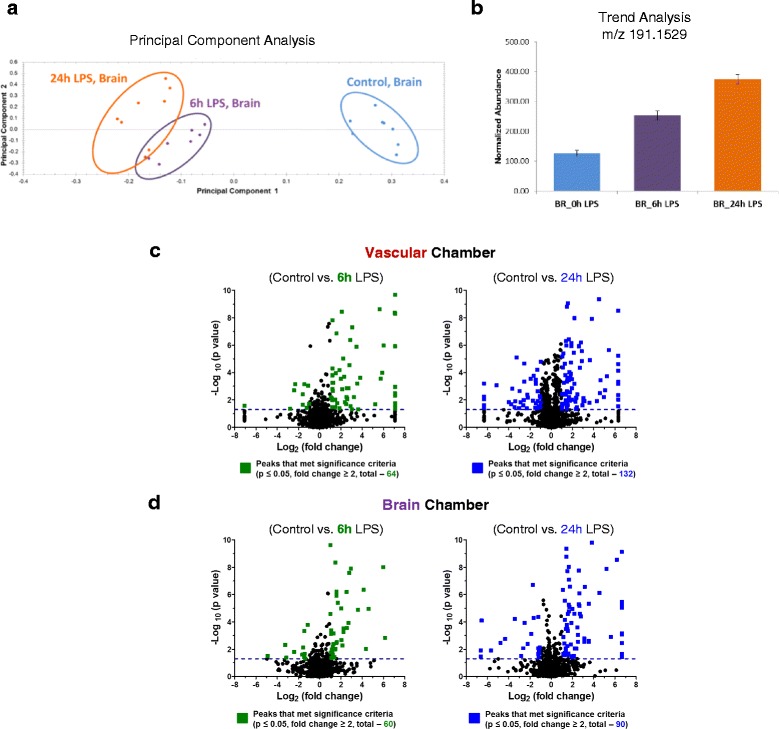
UPLC-IM-MS global metabolomic profile analysis upon LPS treatment. **a** Global principal component analysis (PCA) of LPS-treated (6 and 24 h) and untreated sample types (brain chamber) illustrating that three distinct metabolic signatures or profiles were observed in the UPLC-IM-MS analysis. **b** Trend analysis for m/z 191.1529 illustrates the ability to observe trends in normalized metabolite abundances indicative of treatment exposure times. **c** Volcano plot comparing basal conditions (no LPS treatment) vs.100 μg/mL LPS stimulation in the vascular chamber. In these plots, we observed 64 (6 h) and 132 (24 h) unique compounds that met our significant criteria (fold change ≥│2│ and *p* ≤ 0.05) in the vascular response to LPS. **d** Volcano plot comparing basal conditions (0 LPS) vs.100 μg/mL LPS stimulation in the brain chamber. We observed 60 (6 h) and 90 (24 h) unique metabolites/compounds that met our significant criteria (fold change ≥│2│ and *p* ≤ 0.05) in the brain response to LPS

In addition to looking at the traditional response of our BBB model to inflammatory stimulation in terms of cytokine activation, we also used novel technology to gain a better understanding of how the BBB metabolically responds to these challenges. To investigate the metabolic response of our BBB to inflammatory stimulation over time, UPLC-IM-MS was used to determine if the global molecular metabolic profiles change throughout the course of LPS exposure (see Additional file 1). Principal component analysis plots of the UPLC-IM-MS/MS for LPS samples illustrate distinct separations between the control and treated sample types and between the 6- and the 24-h time points (Fig. 5a, b). Volcano plots for the vascular chamber (Fig. 5c) (*p* ≤ 0.05 and fold changes ≥│2│) illustrate that critical metabolites are released over time (6 h of LPS stimulation; see colored points). We observe the same trend in the brain chamber after 6 h of LPS stimulation (Fig. 5d). Moreover, after 24 h of LPS stimulation, more metabolites that met the significance criteria were released (Fig. 5c, d, right). These global metabolic profile data indicate that metabolic changes occurred after 6 h and before 24 h of stimulation (Fig. 5c, d) in both chambers.

**Fig 6:**
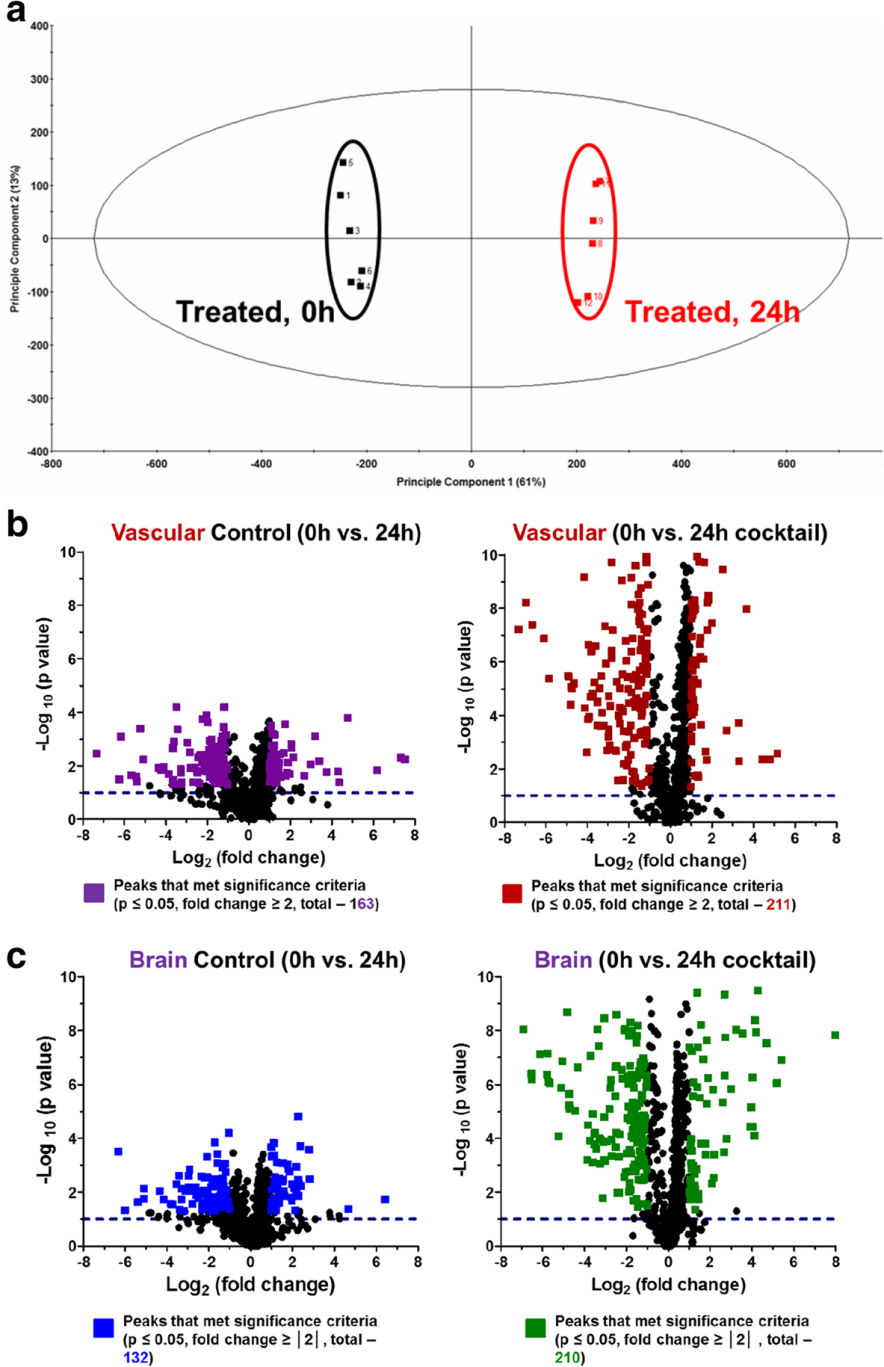
UPLC-IM-MS global metabolomic profile analysis upon cytokine cocktail treatment. **a** Global principal component analysis (PCA) of cytokine cocktail-stimulated (0 and 24 h) sample types (brain chamber) illustrating that two distinct metabolic signatures or profiles were observed in the UPLC-IM-MS analysis. **b** Volcano plot comparing basal conditions (no cytokine cocktail treatment) at 0 and 24 h as well as treated (100 ng/ml cocktail) in the vascular chamber. **c** Volcano plot comparing basal conditions (no cytokine cocktail treatment) at 0 and 24 h as well as treated (100 ng/ml cocktail) in the brain chamber

**Fig. 7:**
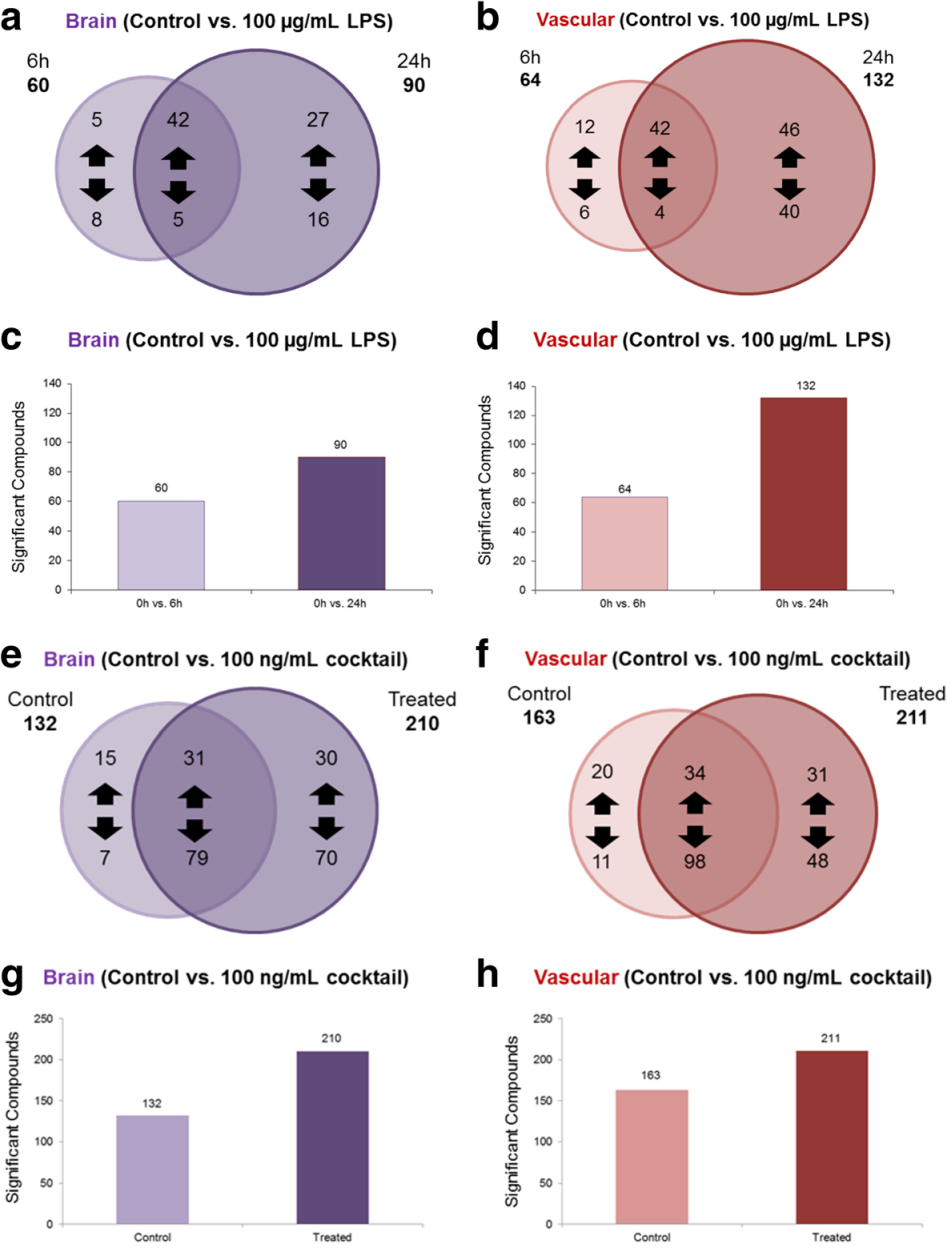
Comparison of metabolites significantly affected by LPS and cytokine cocktail over time in both the vascular and brain chambers. **a** Venn diagram of metabolites/compounds observed for the brain side of the NVU system in response to stimulation with LPS over time (6 and 24 h). **b** Venn diagram of metabolites/compounds observed for the vascular side of the NVU system in response to stimulation with LPS over time (6 and 24 h). **c** Graphical representation of the increase in the total number of features for the brain side. **d** Graphical representation of the increase in the total number of features for the vascular side (significant criteria: *p* ≤ 0.05 and fold change ≥│2│). **e** Venn diagram of metabolites/compounds observed for the brain side of the NVU system in response to stimulation with cytokine cocktail (24 h). **f** Venn diagram of metabolites/compounds observed for the vascular side of the NVU system in response to stimulation with cytokine cocktail (24 h). **g** Graphical representation of the increase in the total number of features for the brain side. **h** Graphical representation of the increase in the total number of features for the vascular side (significant criteria: *p* ≤ 0.05 and fold change ≥│2│)

**Fig. 8:**
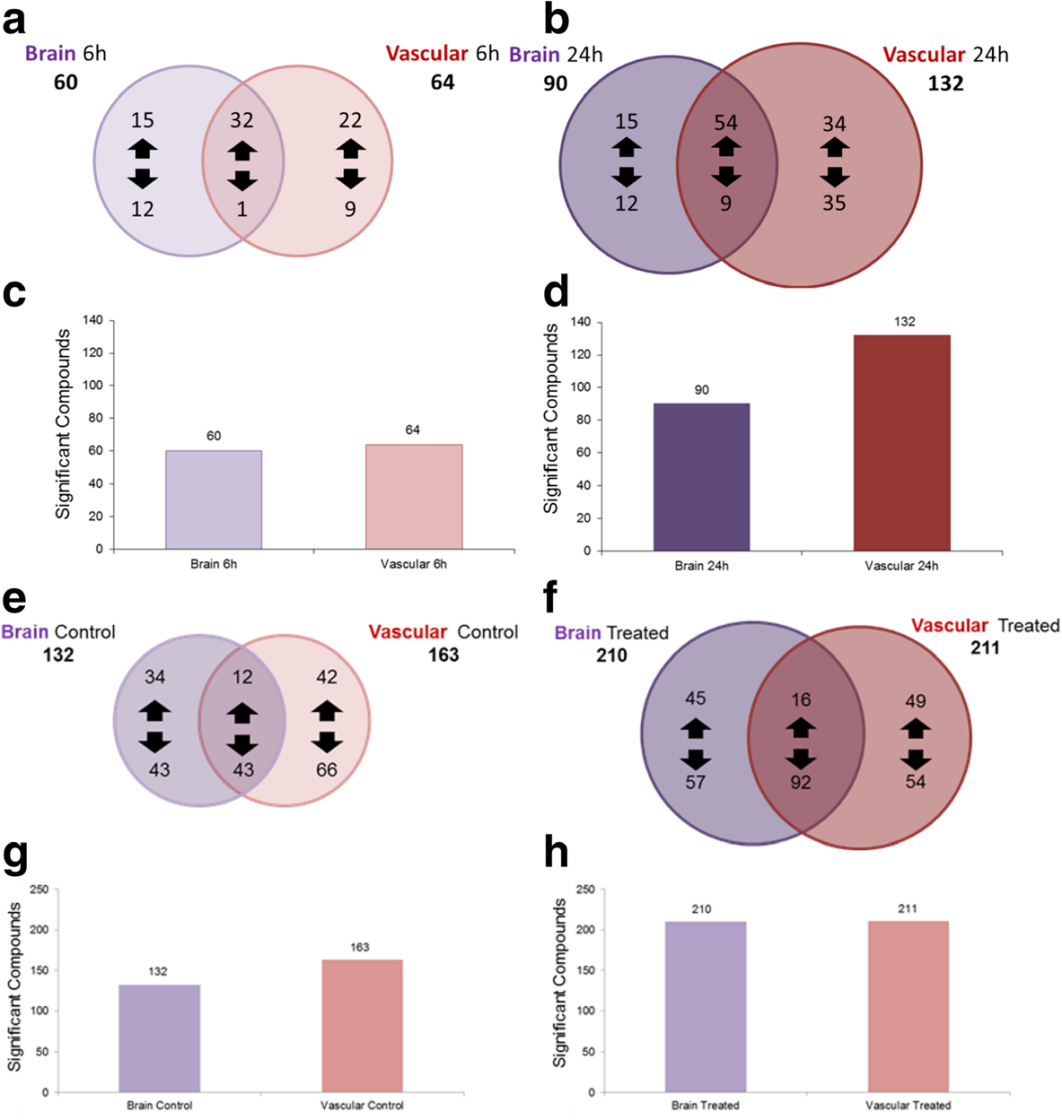
Comparison of the number of metabolites significantly affected by either LPS or cytokine cocktail over time and in both the vascular and brain chambers. **a** Statistically significant metabolites/compounds observed for the brain side and the vascular side of the NVU system in response to stimulation with 100 μg/mL LPS after 6 h of exposure. **b** Statistically significant metabolites/compounds observed for the brain side and the vascular side of the NVU system in response to stimulation with 100 μg/mL LPS after 24 h of exposure. **c** Graphical representation of the increase in the total number of features for the brain side and the vascular side at the 6-h time point. **d** Graphical representation of the increase in the total number of metabolites/compounds for the brain side and the vascular side at the 24-h time point (significant criteria: *p* ≤ 0.05 and fold change ≥│2│). **e** Statistically significant metabolites/compounds observed for the brain side and the vascular side of the NVU system under control conditions. **f** Statistically significant metabolites/compounds observed for the brain side and the vascular side of the NVU system in response to stimulation with 100 ng/mL cytokine cocktail after 24 h of exposure. **g** Graphical representation of the increase in the total number of features for the brain side and the vascular side under control conditions. **h** Graphical representation of the increase in the total number of metabolites/compounds for the brain side and the vascular side at the 24-h time point (significant criteria: *p* ≤ 0.05 and fold change ≥│2│)

In an IM-MS analysis of response to the cytokine cocktail, we observe two distinct groups (0 h treatment and 24 h treatment) using PCA (Fig. 6a). Volcano plots for the vascular chamber (Fig. 6b) (*p* ≤ 0.05 and fold changes ≥│2│) illustrate that both time and treatment have a significant effect on metabolic activity, with treatment causing the biggest impact. We see the same trend in the brain chamber (Fig. 6c). Venn diagrams of the global metabolite profiles show that while a significant number of metabolites are observed after 6 and 24 hours of LPS stimulation, greater than 50% of the compounds observed that met the significance criteria are time-specific (Fig. 7a, b). We do note more significant changes in the global metabolic profiles during the 24-h LPS stimulation when compared to 6 h of stimulation. These observations may be attributed to the cumulative effect of continuous LPS response (Fig. 7c, d). These same trends were consistent with our observations following cytokine cocktail activation (Fig. 7e, f), however, and the inflammatory stimulation was likely a bit stronger given less absorption by the PDMS (Fig. 2a). We found many more metabolites significantly affected by treatment (Fig. 7g, h). The observed global metabolic response to LPS between the vascular and brain chambers (across the BBB) after 6 h of LPS stimulation is similar (see Fig. 8a, b). In contrast, the observed global metabolite profile data for 24 h of LPS stimulation suggest that more significantly changing metabolites were observed in the vascular chamber compared to the brain chamber (Fig. 8c, d). In addition to the global analysis of metabolic trends in response to LPS, a number of significantly changed metabolite compounds were identified through database correlation (see the “Metabolite data processing and analysis” section above, and the tentative structural identifications listed in Additional file 3). These preliminary identifications allow us to prioritize that fatty acid and protein degradation pathways may be affected by LPS stimulation. When examining cytokine cocktail treatment after 24 h of exposure, we see that the overlap between the vascular and brain sides is increased with treatment (Fig. 8e, f), and treatment with cytokine cocktail nearly doubles the number of metabolites changed in both chambers (Fig. 8g, h). Collectively, these two data sets reflecting inflammatory drive show similar but not identical metabolic signatures with regard to the BBB response.

### Pathway identifications of metabolic response to inflammatory stimulation

**Fig. 9:**
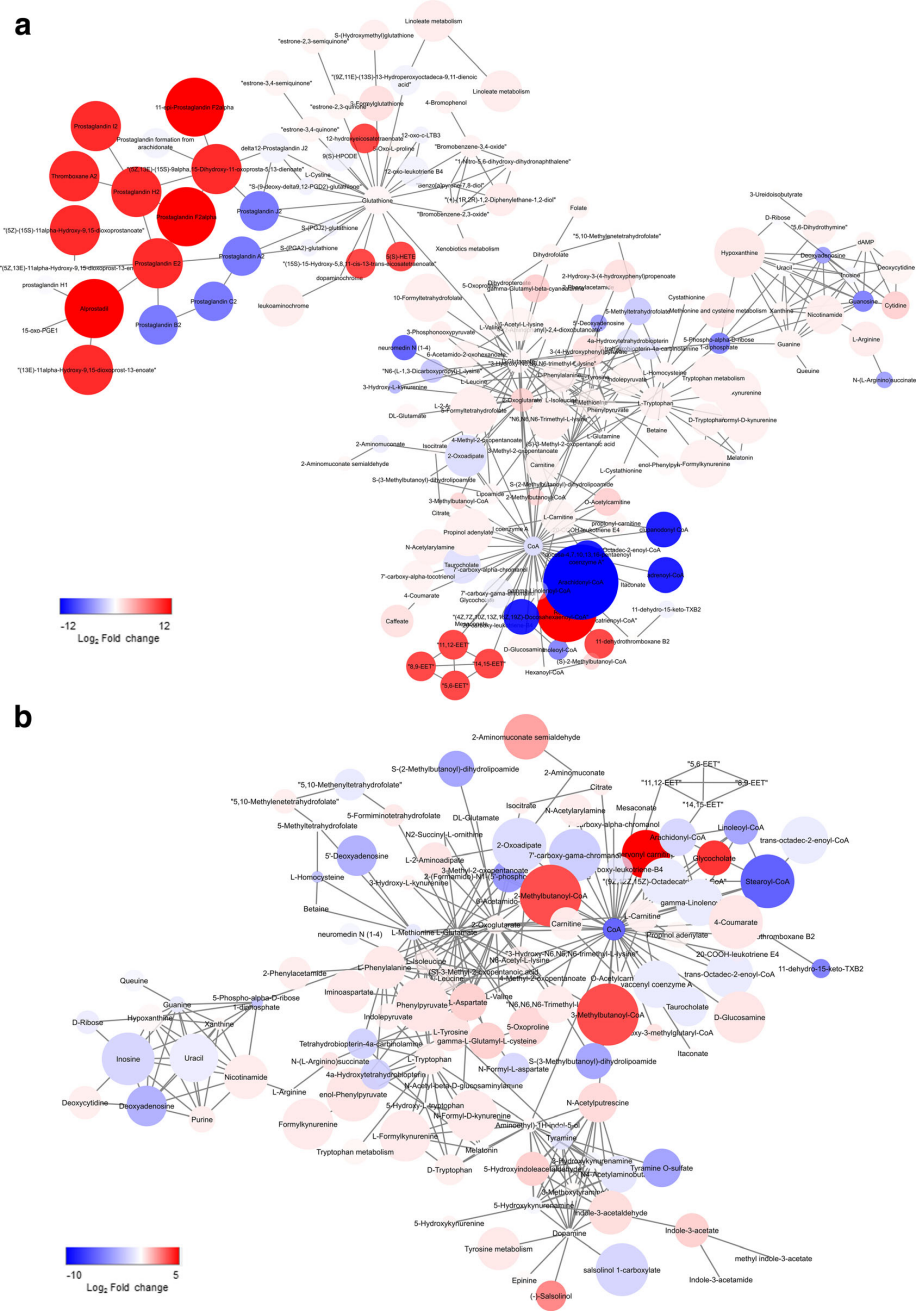
Network module output from mummichog analysis of the qualitative and relative quantitative differences in metabolomic profiles of the response to 100 ng/mL cocktail of IL-1, TNF-α, and MCP-1,2 stimulation for 24 h. Feature m/z values and significance measurements were used to predict metabolic activity networks without the use of conventional MS/MS identification workflows. Metabolites are colored *blue* for negative fold change or *red* for positive fold change, with the color intensity representing the magnitude of fold change and the size representing the statistical significance (−log_10_(*p* value)). **a** Vascular chamber. **b** Brain chamber

**Fig. 10:**
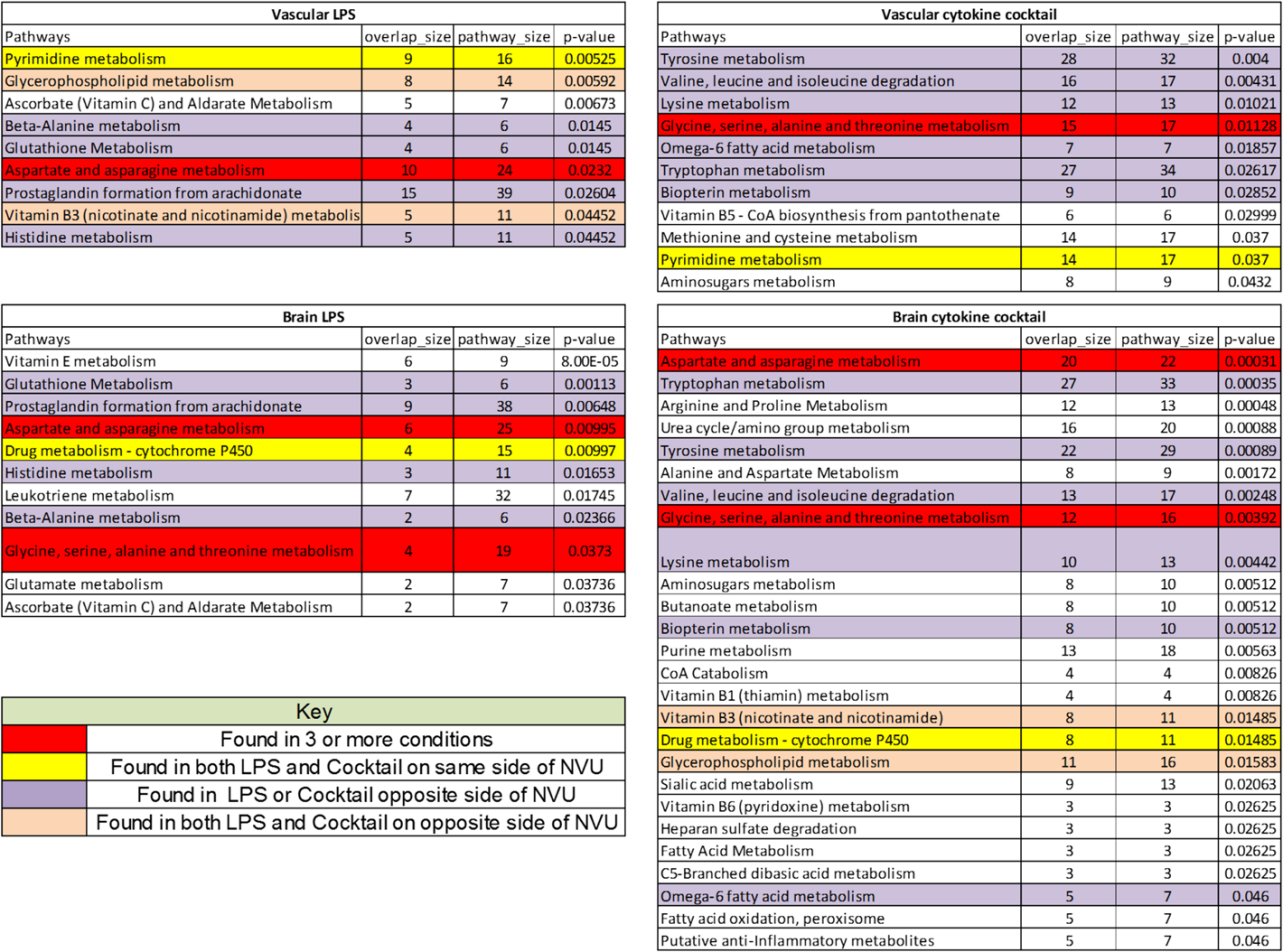
An analysis of the commonalities and differences in pathway activity between LPS and cocktail exposure

Using biologically driven computational analysis (mummichog), metabolites observed in these studies were used to predict metabolic network activity. In determining the BBB response to cytokine cocktail response, network activity analysis (or mummichog) was used to observe metabolites affected either by consumption or production in response to inflammatory stimulation (Fig. 9). These activity network analyses allow our data to be grouped in pathways in an effort to identify network relationships between global metabolic profiles (in this case, treated vs. untreated). In the predictive activity network analysis for the vascular side of the BBB, glutathione, CoA, and tryptophan are highlighted as central nodes (Fig. 9a). On the brain side, CoA and tryptophan are shared central nodes, as are dopamine and glutamate (Fig. 9b). In addition to identifying central nodes of metabolic interconnection, these analyses can also prioritize the significance of specific metabolites in a known pathway and identify specific pathways that are affected by our studies. For these analyses, we used both LPS and cytokine cocktail data sets, and we observed multiple pathways that were continuously highlighted regardless of barrier side or inflammatory stimulation (see Fig. 10, red rows). Glycine, serine, alanine, and threonine metabolism and aspartate and asparagine metabolism indicate that protein synthesis utilizes critical pathways that are changed upon inflammatory stimulation.

We also observed activated pathways specific to each side of the BBB (see Fig. 10, yellow rows). For example, pyrimidine metabolism is a pathway known for being involved with systemic inflammation such as gout and arthritis, as well as some neurodevelopmental disorders [55, 56], and our data suggest its activation in the vascular chamber. These data suggest a novel role for pyrimidine metabolism in BBB response to inflammatory stimulation. In the case of the brain side, the most prominent pathway implicated in our network pathway analysis was drug metabolism involving a canonical cytochrome P450 pathway for mediating the processing of antidepressants, antipsychotics, drugs of abuse, endogenous neurochemicals such as serotonin and dopamine, neurotoxins, and carcinogens [57] and neuroinflammation. There were also several pathways that were treatment-specific (Fig. 10, purple rows). For example, glutathione metabolism was observed for LPS but was not present in the cytokine cocktail data, whereas for the cytokine cocktail treatment, tryptophan metabolism was strongly indicated. It is known that glutathione plays important roles in antioxidant defense, nutrient metabolism, and regulation of cellular events, including cytokine production and immune response, as well as gene expression, DNA and protein synthesis, cell proliferation and apoptosis, and signal transduction. In these data, we observe a broad spectrum of metabolic activity consistent with what we would expect for a compound such as LPS. We also observed some interesting findings, such as the potential activation of tryptophan metabolism, which is critical as an essential amino acid and a key player in serotonin production, but is less known for its effect on inflammation [58].

**Fig. 11:**
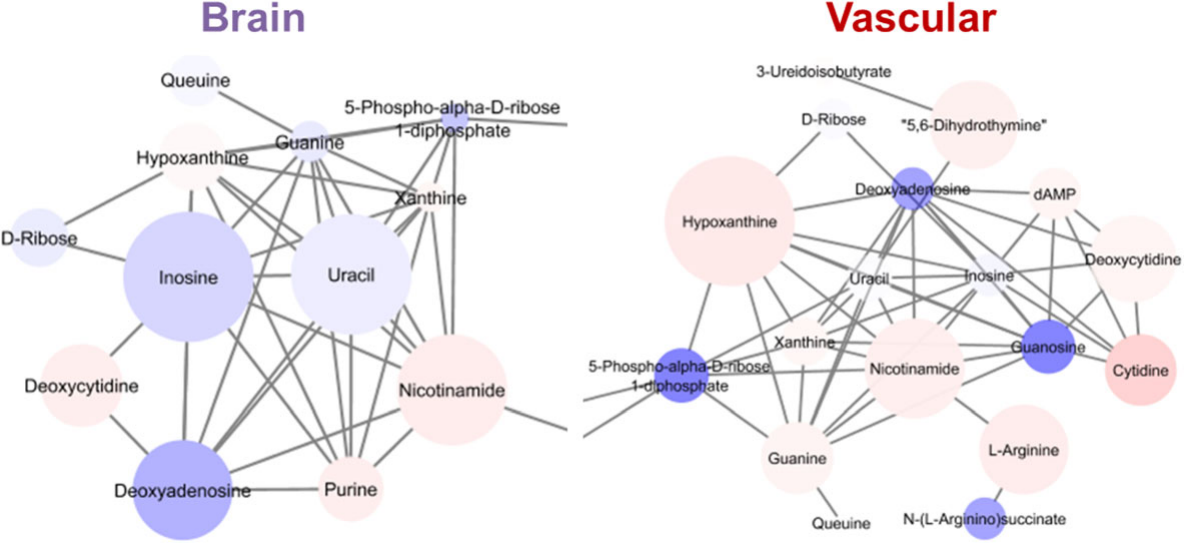
Metabolic activity subnetwork output from mummichog analysis presented in Fig. 9a, b (representative of purine and pyrimidine pathways) for the brain and vascular sides of the NVU stimulated with 100 ng/mL cocktail of IL-1, TNF-α, and MCP-1,2 for 24 h. Feature m/z values and significance measurements were used to predict metabolic activity networks without the use of conventional MS/MS identification workflows. Metabolites are colored blue for negative fold change or red for positive fold change, with the color intensity representing the magnitude of fold change and the size representing the statistical significance ((−log_10_(*p* value))

Network activity analysis also allows us to prioritize the further exploration of networks or metabolites of interest, for example, the purine and pyrimidine pathways (Fig. 10). If we compare activation in the brain side to the vascular side of our BBB model, we observe numerous pathways that are significantly changed in both sides but in opposite directions (Figs. 10 and 11). The vascular chamber is mostly up-regulating the pro-inflammatory pathway, while at the same time, the brain chamber is mostly down-regulating this pathway. By combining a novel micro-organ of a BBB with cutting-edge metabolomics, we have been able to drastically increase our understanding of the metabolic consequences of inflammatory stimulation on the brain microvasculature, as well as the neurons themselves.

## Discussion

In the present study, we report that exposure of the vascular chamber of the NVU to the inflammatory stimulation of either LPS or a cytokine cocktail will induce time-dependent changes in BBB function and cytokine activation, as well as a global change of the metabolomics profile on both the vascular and brain chambers. Our data suggest that the initial response to LPS stimulation is characterized by reduced tight junction formation and increased membrane permeability. While it has been shown that LPS disrupts tight junctions in numerous organ systems, including the gut and lung [59, 60], very few studies have considered its effects on the BBB [61], and none have reported the spontaneous recovery we found: after an increase in exposure time (between 6 and 24 h), a recovery from the initial exposure results in an increase in the formation of tight junctions and a decrease in membrane permeability from the 6-h time point, but not back to the levels before exposure.

In addition to being able to analyze BBB changes in response to LPS and cytokine cocktail, this unique microfluidic model has sufficient cell mass and low enough volume to allow us to conduct cytokine analysis of the effluent from both the vascular and brain sides at the time points chosen. Previous studies have already demonstrated that cytokines are often released in an oscillating fashion and may have both pro- and anti-inflammatory properties, depending on their release profiles [62–64]. The 3D cell culture made possible by our NVU [23] enabled us to see and profile cytokine activation based not only on when cytokines were elevated but also on whether they were differentially elevated in terms of vascular vs. brain compartment. Taken collectively, these data argue for a cytokine activation model whereby pro-inflammatory cytokines are activated in both chambers at initial exposure, but at later time points, only a subset remains activated in both compartments. The data also suggest that at these later time points, pro-repair cytokine activation is up-regulated in the brain compartment as the BBB begins to rebound, whereas the vascular compartment remains more pro-inflammatory.

Notable advances in identifying metabolites and understanding the significance of these measurements are demonstrated in these studies. By leveraging IM-MS with our organ-on-chip model of the BBB, we were able to obtain a more thorough investigation of metabolite changes due to neuroinflammation. In these studies, our data suggest that (1) inflammation involving the BBB is closely linked to protein synthesis, (2) different sides of the barrier use different proteomic and metabolic pathways to respond to inflammatory signals, and (3) even when the same pathway is involved, the vasculature could be driving pro-inflammatory processes while the brain is ramping down inflammation.

## Conclusions

This work integrated several new technologies, including microfluidic organs-on-chips as in vitro models, IM-MS metabolomics, and pathway identification, and it is from this integration that we have gained new insights into BBB response to inflammatory stimulation. We have shown inflammatory disruption of the BBB and transport of the inflammatory signals across the BBB, mapped changes in cytokine to barrier disruption, and created a detailed analysis of the metabolic signature and metabolic pathways associated with inflammatory stimulation.

## Additional files

Additional file 1: Figure S1. Experimental design and sample workflow. (PDF 782 kb)
Additional file 2: Table S1. Cytokines measured with ELISA. (PDF 391 kb)
Additional file 3: Table S2. Tentative structural identifications. (XLSX 69 kb)

## Abbreviations

ANOVA: Analysis of variance
BBB: Blood-brain barrier
CNS: Central nervous system
ELISA: Enzyme-linked immunosorbent assay
FITC-dextran: Fluorescein isothiocyanate-dextran
GM-CSF: Granulocyte-macrophage colony-stimulating factor
hBMVEC: Human brain-derived microvascular endothelial cells
HDMS^E^: High-definition data-independent mass spectrometry acquisition with simultaneous analysis of molecular fragmentation
hiPSC: Human induced pluripotent stem cells
IM-MS: Ion mobility-mass spectrometry
LC: Liquid chromatography
LPS: Lipopolysaccharide
MS: Mass spectrometry
MS^E^: Data-independent MS acquisition with simultaneous analysis of molecular fragmentation
NVU: NeuroVascular Unit
PCA: Principal component analysis
PDMS: Polydimethylsiloxane
ROCK: Rho-associated coiled-coil kinase
TEER: Transendothelial electrical resistance
UPLC: Ultraperformance liquid chromatography
VIIBRE: Vanderbilt Institute for Integrative Biosystems Research and Education
