# Supplementary figures and images for: Metabolic consequences of inflammatory disruption of the blood-brain barrier in an organ-on-chip model of the human neurovascular unit
Source: J Neuroinflammation. 2016 Dec 12;13:306. doi: 10.1186/s12974-016-0760-y (PMC5153753; doi:10.1186/s12974-016-0760-y)

Figure S1.

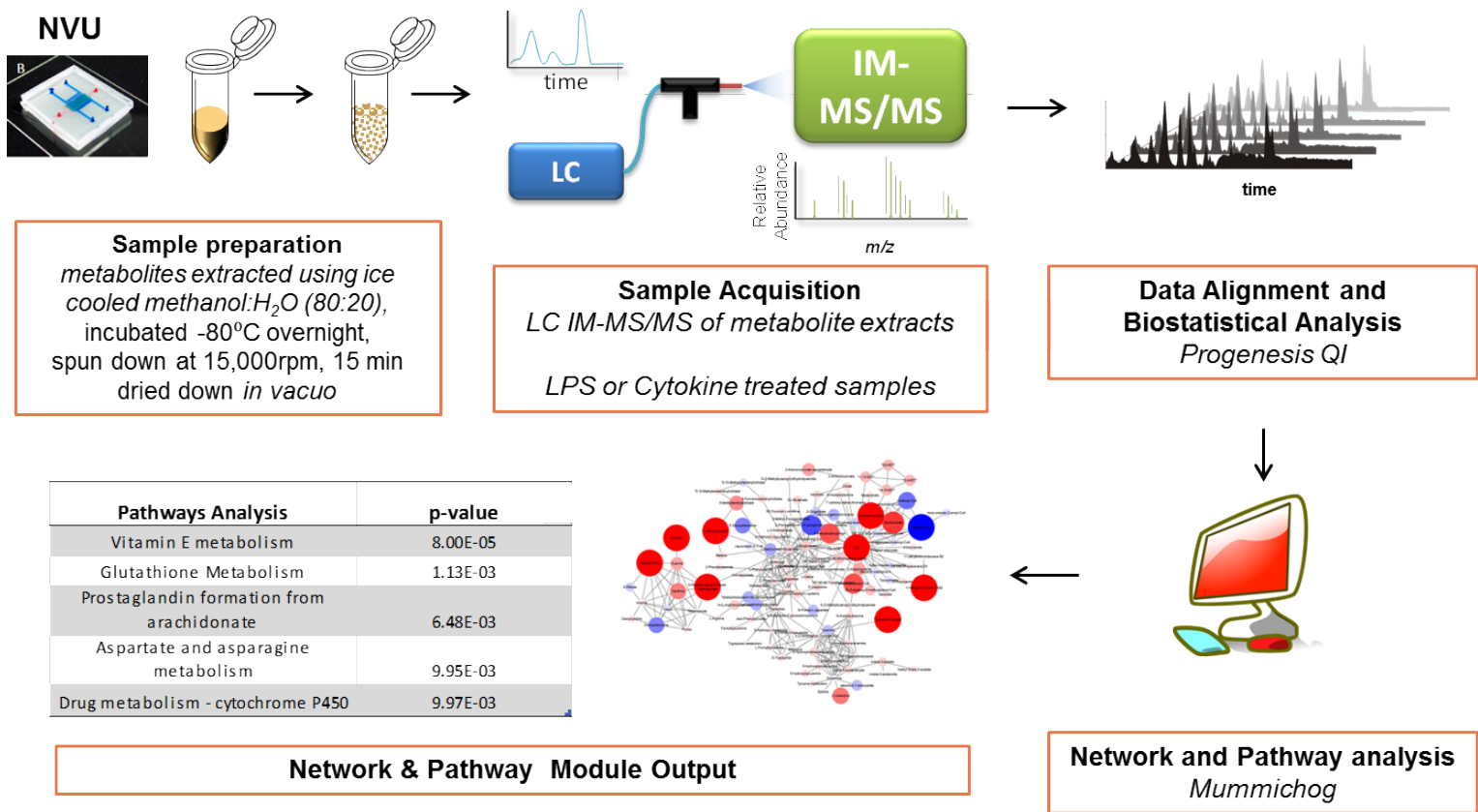

Figure S1. Experimental design and sample workflow

Supplement: Additional file 1: Figure S1. — Experimental design and sample workflow. (PDF 782 kb) [file 12974_2016_760_MOESM1_ESM.pdf]
