# Supplementary material for: Metabolic consequences of inflammatory disruption of the blood-brain barrier in an organ-on-chip model of the human neurovascular unit
Source: J Neuroinflammation. 2016 Dec 12;13:306. doi: 10.1186/s12974-016-0760-y (PMC5153753; doi:10.1186/s12974-016-0760-y)
Supplement: Additional file 2: Table S1. — Cytokines measured with ELISA. (PDF 391 kb) [file 12974_2016_760_MOESM2_ESM.pdf]

Table S1.

Cytokine		Vascular Chamber		Brain Chamber	
		6 hours	24 hours	6 hours	24 hours
GM_CSF	fold Δ	8	2	3	-3
	P-val	0.02	N.S.	0.016	N.S.
IL12-p40	fold Δ	4	0	0	0
	P-val	0.0047	N.S.	N.S.	N.S.
IL-15	fold Δ	0	0	2	0
	P-val	N.S.	N.S.	N.S.	N.S.
IL-16	fold Δ	2	2	2	2
	P-val	0.0067	0.05	N.S.	N.S.
IL-17A	fold Δ	-3	-2	6	4
	P-val	0.05	0.05	0.001	0.002
IL-1a	fold Δ	1	1	1	1
	P-val	N.S.	N.S.	N.S.	N.S.
IL-5	fold Δ	1	1	3	2
	P-val	N.S.	0.02	0.02	N.S.
IL-7	fold Δ	2	1	2	2
	P-val	N.S.	N.S.	0.03	N.S.
TNF-b	fold Δ	0	-0.5	2	2
	P-val	N.S.	N.S.	N.S.	N.S.
VEGF	fold Δ	-2	-4	2	1
	P-val	N.S.	N.S.	N.S.	N.S.
TNF-α	fold Δ	1.5	3	2	3
	P-val	0.01	0.01	0.01	0.01
IL-1b	fold Δ	0	2	1.5	2
	P-val	N.S.	0.01	N.S.	0.001
